# CD4^+^ T-cell subsets in autoimmune hepatitis: A review

**DOI:** 10.1097/HC9.0000000000000269

**Published:** 2023-09-11

**Authors:** Haoran Chen, Zhongyu Han, Yiyue Fan, Liuyan Chen, Fang Peng, Xuhua Cheng, Yi Wang, Junyan Su, Dongxuan Li

**Affiliations:** 1 Chengdu Xinhua Hospital, Chengdu, China; 2 School of Medical and Life Sciences, Chengdu University of Traditional Chinese Medicine, Chengdu, China; 3 Affiliated Hospital of North Sichuan Medical College, Nanchong, China; 4 The First People’s Hospital of Longquanyi District, Chengdu, China

## Abstract

Autoimmune hepatitis (AIH) is a chronic autoimmune liver disease that can lead to hepatocyte destruction, inflammation, liver fibrosis, cirrhosis, and liver failure. The diagnosis of AIH requires the identification of lymphoblast cell interface hepatitis and serum biochemical abnormalities, as well as the exclusion of related diseases. According to different specific autoantibodies, AIH can be divided into AIH-1 and AIH-2. The first-line treatment for AIH is a corticosteroid and azathioprine regimen, and patients with liver failure require liver transplantation. However, the long-term use of corticosteroids has obvious side effects, and patients are prone to relapse after drug withdrawal. Autoimmune diseases are characterized by an imbalance in immune tolerance of self-antigens, activation of autoreactive T cells, overactivity of B cells, and increased production of autoantibodies. CD4^+^ T cells are key players in adaptive immunity and can secrete cytokines, activate B cells to produce antibodies, and influence the cytotoxicity of CD8^+^ T cells. According to their characteristics, CD4^+^ T cells can be divided into different subsets. In this review, we discuss the changes in T helper (Th)1, Th2, Th17, Th9, Th22, regulatory T cell, T follicular helper, and T peripheral helper cells and their related factors in AIH and discuss the therapeutic potential of targeting CD4^+^ T-cell subsets in AIH.

## INTRODUCTION

Autoimmune hepatitis (AIH) is an immune-mediated non–self-limiting liver disease that can lead to hepatocyte destruction, inflammation, hepatic fibrosis, cirrhosis, and liver failure.^1^ AIH occurs in different human populations of all ages, with an incidence rate of ~4–42.9/100,000, among which women aged 10–30 and 40–60 have the highest incidence rates.^2^ AIH has no characteristic diagnosis, and clinical diagnosis of AIM requires characteristic histological abnormalities (interfacial hepatitis), elevated laboratory indicators [serum alanine aminotransferase (ALT), serum aspartate aminotransferase, serum IgG, antinuclear antibodies (ANAs), smooth muscle antibodies (SMAs), and antibodies to liver-kidney microsome type 1 (anti-LKM1) levels], and exclusion of various liver diseases and liver injuries similar to AIH, such as viral hepatitis and DILI.^2^ According to different specific autoantibodies, AIH is divided into AIH-1 (ANA^+^, SMA^+^, SLA^+^, anti-actin^+^) and AIH-2 (anti-LKM1^+^, anti-LC1, anti-LKM3).^3,4^ In addition, ~20% of the patients were negative for these antibodies, which is called serum-negative AIH.^2^ At present, the first-line treatment for AIH is a corticosteroid and azathioprine regimen, which aims to improve the symptoms of patients, control inflammation, and achieve biochemical remission; mycophenolate mofetil, tacrolimus, and cyclosporine are also normally used in the treatment of AIH patients. Patients with acute severe AIH leading to liver failure require liver transplantation.^5^ However, long-term use of corticosteroids has obvious side effects, and patients are prone to relapse after drug withdrawal.^6^ Therefore, it is necessary to study the pathogenesis of AIH in depth to provide evidence for the diagnosis and treatment of AIH.

**FIGURE 1:**
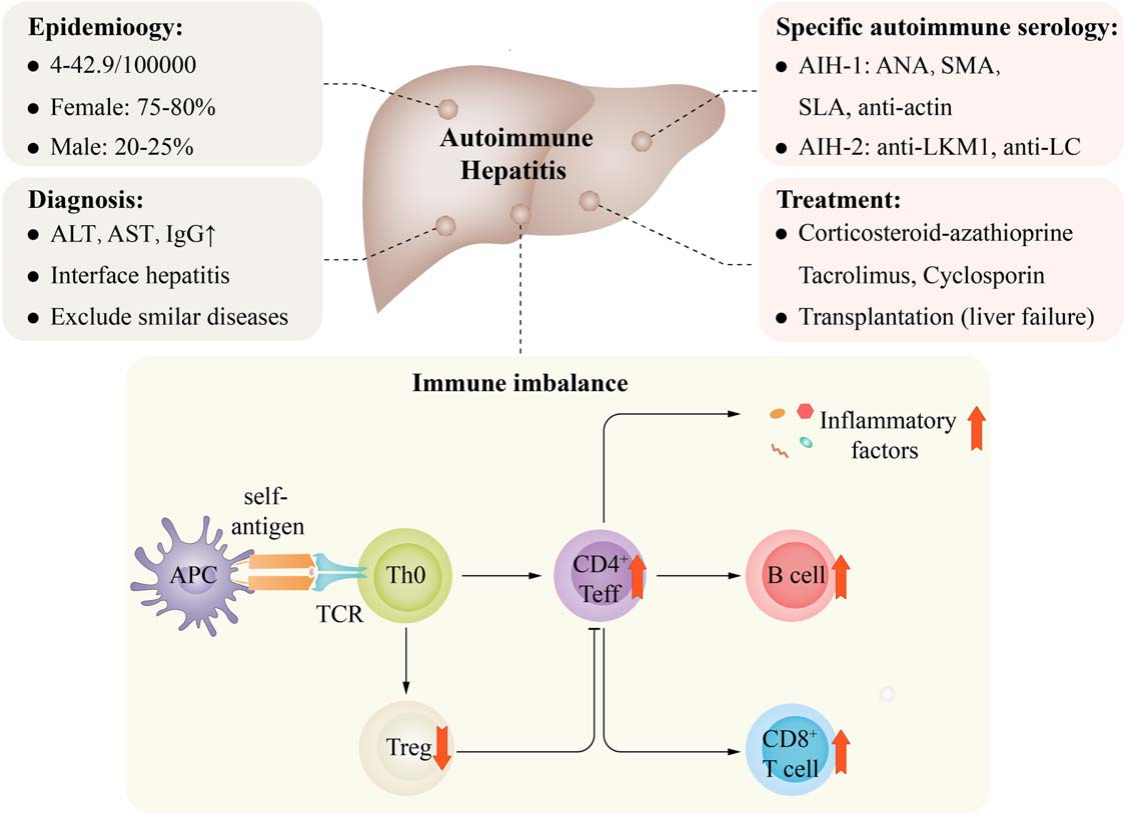
Epidemiology, diagnosis, autoimmune serology, treatment, and mechanisms of autoimmune imbalance in AIH. Abbreviations: AIH, autoimmune hepatitis; ALT, alanine aminotransferase; ANA, antinuclear antibody; APC, antigen-presenting cell; AST, aspartate aminotransferase; LKM1, liver kidney microsome type 1; SLA, soluble liver antigen; SMA, smooth muscle antibody; Treg, regulatory T cell.

Autoimmune diseases are characterized by an imbalance in immune tolerance to self-antigens, leading to abnormal immune active and autoimmune attacks on target organs.^7^ Similar to other autoimmune diseases, AIH is characterized by the activation of autoreactive T cells, overactivity of B cells, and increased autoantibody production.^8^ CD4^+^ T cells (or T helper cells, Th cells) are key players in adaptive immunity. They can secrete cytokines, activate B cells to produce antibodies, and affect the cytotoxicity of CD8^+^ T cells.^9^ Abnormal CD4^+^ T cells can lead to serious autoimmune disease, and significant CD4^+^ T-cell infiltration can be observed in the liver of AIH patients (Figure 1). The genetic susceptibility of human leukocyte antigen-II (HLA-II) alleles in AIH and the liver infiltration of CD4^+^ T cells in AIH indicate the key role of CD4^+^ T cells in AIH.^1^ In this review, we focus on recent research and investigations on the differentiation of different CD4^+^ T-cell subsets in AIH and the expression of their related factors and discuss the therapeutic potential of targeting CD4^+^ T-cell subsets in AIH.

## DIFFERENTIATION OF CD4^+^ T-CELL SUBSETS

In addition to thymic-induced regulatory T cells (tTregs), other CD4^+^ T subsets are differentiated from naive CD4^+^ T cells (Th0), whereas tTregs and Th0 cells are differentiated from CD4^+^CD8^+^ cells in the thymus.^10^ In the thymus, CD4^+^CD8^+^ T cells undergo positive and negative selection to differentiate and select Th0 cells that bind to class II major histocompatibility complex (MHC II) with moderate affinity and then leave the thymus.^11–14^ After entering the periphery, Th0 cells recognize the cognate antigen presented by professional antigen-presenting cells (APCs), such as macrophages, dendritic cells, and B cells.^15,16^ APCs have HLA-II molecules on the surface of membranes, which bind to peptide chains that need to be recognized.^17^ When peptides are recognized as non-self, Th0 cells are activated and differentiated into different CD4^+^ T-cell subsets. At present, the identified CD4^+^ T-cell subsets mainly include Th1, Th2, Th17, Th9, and Th22 cells, regulatory T cells (Tregs), T follicular helper (Tfh) cells, and T peripheral helper (Tph) cells (Table 1).

**TABLE 1:** CD4^+^ T-subsets differentiation, signature cytokines, and changes in AIH

CD4^+^ T-cell subsets	Differentiation	Transcription factors	Production	Changes in AIH	References
Th1	IL-12	T-bet	IFN-γ, TNF-α, IL-2, CXCR3	Increased Th1 differentiation and liver infiltration	^18–25^
Th2	IL-4, IL-2	GATA3	IL-4, IL-5, IL-13, IL-10	Increased or remained unchanged Th2 and imbalanced Th1/Th2 ratio	^18,26–31^
Th17	IL-6, IL-21, TGF-β, IL-1β, IL-23	RORγt	IL-17, IL-21, IL-22, CCR4, CCR6	Th17-related factors increased in the liver, Th17 infiltration increased, CD39^+^ Th17 decreased	^32–38^
Treg	TGF-β, IL-2	Foxp3	TGF-β, IL-10, CD25, CTLA-4, CD39	Increased or decreased Treg and decreased Treg/CD4^+^ Teff ratio	^39–45^
Th9	IL-4, TGF-β	PU.1, IRF4	IL-9, IL-10	Increased IL-9 levels associated with liver fibrosis and cirrhosis	^46^
Th22	IL-6, TNF-α, IL-1β, IL-21, IL-23	AhR	IL-22, CCR4, CCR6, CCR10, TNF-α, IL-13	AhR and IL-22 levels increased, which could improve Treg/CD4^+^ Teff ratio	^47–50^
Tfh	IL-21, IL-6, IL-12, Activin A, ICOS	Bcl-6, c-Maf	IL-21, PD-1, CXCR5	Increased IL-21^+^ICOS^+^CXCR5^+^ Tfh positively correlated with the severity of AIH	^51–56^
Tph	IL-21, IL-6, IL-12, ICOS	Blimp-1, c-Maf, Sox4	IL-21, PD-1, CXCL13	Increased IL-21^+^ICOS^+^CXCL5^−^ Tph mainly stimulated humoral immunity in AIH	^57^

Abbreviations: AhR aryl hydrocarbon receptor; AIH, autoimmune hepatitis; Bcl-6, B-cell lymphoma 6; Blimp-1, B lymphocyte maturation protein 1; CCR, C-C chemokine receptor; c-Maf, cellular musculoaponeurotic fibrosarcoma; CTLA-4, cytotoxic T-lymphocyte-associated antigen-4; CXCL13, CXC chemokine ligand 13; CXCR, CXC chemokine receptor; Foxp3, forkhead box P3; GATA3, gata-binding protein-3; GM-CSF, granulocyte-macrophage colony-stimulating factor; ICOS, inducible costimulatory; IFN-γ, interferon gamma; IRF4, interferon regulatory factor 4; PD-1, programmed cell death protein 1; RORγT, retinoid-related orphan receptor-gamma t; Sox4, SRY-related high mobility group box 4; Teffs, effector T cells; Tfh, T follicular helper; Th, T helper; Tph, T peripheral helper; Treg, regulatory T cell.

### Th1 cells and AIH

**FIGURE 2:**
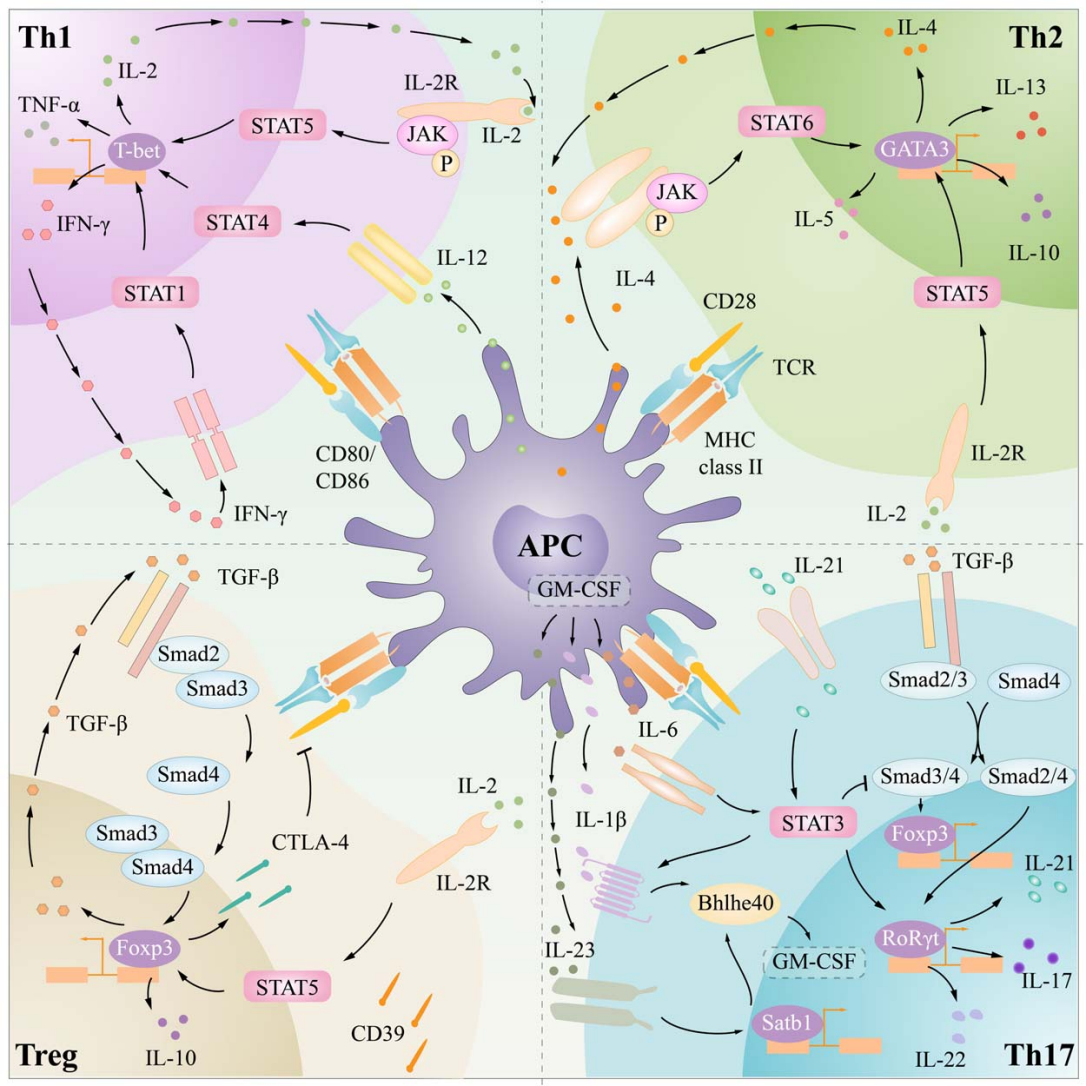
Processes of Th1, Th2, Th17, Treg differentiation. pMHC-TCR and CD28-CD80/CD86 costimulation and various cytokines induced different differentiation of Th0. IL-2, IL-12, and IFN-γ promote T-bet expression and Th1 differentiation. IL-2 and IL-4 promote GATA3 expression and Th2 differentiation. TGF-β, IL-21, IL-6 promote RORγt expression and Th17 differentiation, while IL-1β and IL-23 promote pathogenic Th17. TGF-β and IL-2 induced Foxp3 expression and Treg differentiation. Abbreviations: APC, antigen-presenting cell; Foxp3, forkhead box P3; GATA3, gata-binding protein-3; GM-CSF, granulocyte-macrophage colony-stimulating factor; IFN-γ, interferon gamma; JAK, Janus kinase; MHC, major histocompatibility complex; RORγT, retinoid-related orphan receptor-gamma t; STAT, signal transducer and activator of transcription; TYK, tyrosine kinase.

Th1 cells are characterized by the expression of the transcription factor T-bet and the production of the cytokine interferon gamma (IFN-γ).^58^ In addition, Th1 cells produce various cytokines, such as IL-2, IL-10, TNF-α, and CXC chemokine receptor 3 (CXCR3).^59^ When Th0 recognizes APC-pHLA, IL-12 can promote T-bet expression, Th1 conversion, and IFN-γ generation through the IL-12-signal transducer and activator of transcription 4 (STAT4) pathway.^60^ T-bet is encoded by *Tbx21* and is a key transcription factor in Th1 cell differentiation.^61^ DNase I hypersensitivity site sequencing identified that the transcription-start site 12 kb upstream of *Tbx21* (*Tbx21-CNS-12*) contains STAT binding motifs.^62^ *Tbx21-CNS-12* can also be accessible in Th0 cells, which indicates that it can react to cytokines in the Th0 stage.^63^ Studies have shown that the IL-12-STAT4, IFN-γ-STAT1, and IL-2-STAT5 axes can upregulate the expression of T-bet and promote Th1 differentiation.^64,65^ Among these factors, IFN-γ and IL-2 can be produced by Th1 cells, and positive feedback enhances Th1 differentiation (Figure 2).

Th1 cells play important roles in the pathogenesis of AIH. Behfarjam et al^18^ found that the levels of T-bet, IFN-γ, and TNF-α were significantly increased in the blood of untreated AIH patients. TNF-α can play pathogenic or protective roles in AIH, which can lead to apoptosis or differentiation of hepatocytes.^19^ Chemokine (C-C motif) ligand 20 (CCL20) mediates the migration of a variety of immune cells to the liver by binding to its specific receptor C-C chemokine receptor 6 (CCR6).^20^ Iwamoto et al^66^ found that TNF-α could induce an increase in hepatic CCL20 expression and play a pathogenic role in the concanavalin-A (Con A)-induced AIH model. IFN-γ can promote IgG2a antibody conversion and MHC I and MHC II antigen presentation and activate a variety of cells.^67^ CXCR3 is highly expressed in Th1 cells, CTLs, natural killer (NK) cells, and other cells, and its ligand CXC chemokine ligand (CXCL)9-11 can be induced by IFN-γ produced by Th1 cells.^68^ The CXCL9-11/CXCR3 axis can lead to the recruitment of Th1 cells, CTLs, and NK cells to inflamed sites, leading to inflammatory cell infiltration in the liver.^69,70^ Bovensiepen et al^21^ found that TNF-α-producing Th1 cells were significantly expanded in peripheral blood mononuclear cells (PBMCs) and liver of AIH patients and that IFN-γ expression was also elevated in the liver. Yang et al^22^ used methyl butyrate to inhibit Th1 differentiation and homing to achieve a therapeutic effect in the Con A-induced AIH model. In their model, ALT and aspartate aminotransferase levels were significantly elevated, and inflammatory cell infiltration in the liver was evident. The frequency of Th1 cells was significantly increased, as was the expression of IFN-γ, TNF-α, CXCR3, and CXCL9-11. After Th1 cells were inhibited, the above indices were significantly improved.^22^ Gil-Farina et al^23^ created mice with transient IL-12 transgene expression, which resulted in a persistent elevation in IL-12 and IFN-γ expression and induction of AIH-1-like chronic hepatitis. Mix et al^24^ found that the autoantigen peptides in AIH-specific SLA/LP could be targeted by CD4^+^ T cells to produce IFN-γ, and are regulated by the AIH susceptibility gene HLA-DRB1*0301. AIH-2 patient characteristic antibody anti-LKM1 recognizes cytochrome P450 IID6. Studies have shown that cytochrome P450 IID6 can damage hepatocytes by promoting specific Th1 cells to produce IFN-γ and produce autoantibodies.^25^ A Chinese study showed that STAT4 polymorphisms were positively associated with AIH-1 in Chinese Han children,^71^ but a Tunisian study showed that STAT4 polymorphisms were not associated with AIH in the local population, which may be related to a difference in susceptibility genes among different populations.^72^

### Th2 cells and AIH

Th2 cells are characterized by the expression of gata-binding protein-3 (GATA3) and the production of IL-4, IL-5, IL-6, and IL-13.^58^ When Th0-TCR recognizes APC-pHLA, the IL-12-STAT4 axis guides Th1 differentiation, while the IL-4-STAT6 axis mediates Th2 differentiation.^73^ However, IL-4 or STAT6 alone is not the determinant of Th2 differentiation. Studies have shown damaged Th2 differentiation in IL-4-STAT6-deficient mice, but under Th1-differentiating conditions, the enforced expression of GATA3 by reverse transcription can lead to Th2 differentiation in IL-4-deficient mice.^74,75^ Subsequent studies have shown that GATA3 plays a major regulatory role in Th2 cell differentiation. GATA3^−^ Th0 cells show impaired Th2 differentiation and can undergo Th1 differentiation in the absence of IL-12 and IFN-γ.^76,77^

GATA3 can be activated by the IL-4-STAT6 and IL-2-STAT5 axes. In the IL-4-STAT6 pathway, IL-4-IL-4R binding can activate the Janus kinase (JAK)-STAT pathway and induce the phosphorylation and dimerization of STAT6.^78^ Then, STAT6 enters the nucleus and activates GATA3 to promote Th2 differentiation.^79^ GATA3 can promote the production of cytokines such as IL-4, IL-5, and IL-13, and IL-4-GATA3 positive feedback maintains Th2 polarization and production of Th2 memory cells. In addition, GATA3 can inhibit IFN-γ and STAT4 to inhibit Th1 differentiation.^80^ STAT5 is also important in Th2 cell differentiation. Studies have shown that Th2 differentiation is impaired in STAT5a− mice; compared with STAT6− mice, STAT5−STAT6− mice show further impairments in Th2 cell differentiation.^73,81^ Under Th1-differentiating conditions, reverse transcription of STAT5a can induce Th2 differentiation.^81^ However, reverse transcription of STAT5a cannot induce Th2 differentiation in GATA3− mice, which emphasizes the role of GATA3.^76^ IL-2 is mainly produced by activated CD4^+^ T cells and is the most effective inducer of STAT5 activation.^64^ IL-2-mediated STAT5 signal transduction is necessary for Th2 cell differentiation in vitro. The signal transduction activated by IL-2 is related to the intensity of the TCR signal. Low-intensity TCR stimulation leads to the production of IL-2 and upregulation of IL-2Ra, while strong TCR stimulation inhibits IL-2 signaling.^82,83^ IL-2 activates the STAT5-GATA3 axis, resulting in the production of IL-4. This IL-4 production depends on only the activation of TCR, not on IL-4 in the environment. In addition, GATA3 and STAT5 can regulate the expression of IL-2R and IL-4R to promote IL-4-GATA3 positive feedback.^83^ Multiple pathways, such as the Notch pathway and Wnt-β-Catenin pathway, may regulate the expression of GATA3 and Th2 differentiation.^84,85^ Th2 cells produce cytokines such as IL-4, IL-5, IL-13, IL-9, and IL-10, which can induce B cells to produce IgG1 and IgE, activate M2 macrophages, and recruit eosinophils.^86^ Th2 cells play roles in infection, helminth infection, and allergies and are also related to autoimmune diseases (Figure 2).

Cytokines produced by Th2 cells can counteract the proinflammatory effects of Th1 cells and promote humoral immunity.^73^ IL-4 can promote B-cell differentiation and induce antibody class switching to IgG1 and IgE.^87^ IL-5 can stimulate the activation of B cells to produce antibodies and the maturation of eosinophils.^88^ IL-13 can promote the maturation of B cells and inhibit proinflammatory cytokines, such as IL-12 and TNF-α.^89^ Th1/Th2 imbalance can cause a variety of autoimmune diseases.^26,90,91^ Behfarjam and colleagues found that T-bet and IFN-γ levels were significantly elevated in PBMCs of AIH patients, but the expression of GATA3 and IL-4 of the patients was not significantly different from that of controls, indicating a Th1/Th2 imbalance.^18,27^ In another study, CD4^+^CD25^+^IL-4^+^ cells showed no significant changes in AIH, AIH-systemic lupus erythematosus (SLE)/MCTD, and healthy controls.^28^ A protein array study showed high specificity and sensitivity of IL-4 in AIH, and inhibition of IL-4-STAT6 was detected in the serum of AIH patients.^29^ Yousefi et al^30^ reported that the IL-4-33 TT genotype and IL-4-590 C/T polymorphism were susceptibility genes in Iranian AIH patients. Kawashima et al^31^ reported a significant increase in the IFN-γ concentration in PBMCs of children with AIH but no significant change in that of IL-4. Zachou et al^92^ found a significant increase in the levels of the Th2-related cytokines IL-4 and IL-10 and the Th1-related cytokines IFN-γ and TNF-α in the bone marrow of patients with AIH-1, but they did not measure the Th1/Th2 ratio. IL-4 and specific antibodies were increased in cytochrome P450 IID6–induced AIH-2.^25^ Chi et al^93^ induced AIH using TLR2/4 ligand-amplified liver inflammation and found increased expression levels of the *IL4* and *IL13* genes and IL-4 and IL-13 proteins in the liver. However, this Th2 response has an insufficient inhibitory effect on Th1 cells, which is conducive to the maturation and humoral immune function of B cells, highlighting the pleiotropy of Th2 cells in AIH.^93^

### Th17 cells and AIH

Th17 cells express IL-1R, IL-23R, and chemokine receptors CCR4 and CCR6, and can produce IL-17A, IL-17F, IL-21, IL-22, and TNF-α. Retinoid-related orphan receptor-γt (RORγt) is a major regulator of Th17 differentiation.^94^ Th17 differentiation requires IL-6, TGF-β, and IL-1β in humans and IL-6, TGF-E, and IL-21 in mice.^95^ TGF-β-Smad pathway plays an important role in Th17 and Treg differentiation.^96^ TGF-β combines with TGF-βR to promote the phosphorylation of Smad2 and Smad3, and then p-Smad2 and p-Smad3 bind with Smad4 to regulate the expression of RORγt and forkhead box P3 (Foxp3), respectively.^96,97^ IL-6 activates RORγt and leads to the production of IL-17 through the IL-6-JAK2-STAT3 pathway.^98^ IL-6 can also inhibit the TGF-β-mediated induction of Foxp3 to regulate Th17/Treg balance.^99^ IL-2 plays a major negative regulatory role in Th17 differentiation.^100^ On the one hand, Th17 cells can secrete inflammatory factors, recruit neutrophils, and play a proinflammatory role. On the other hand, Th17 cells can also produce cytokines such as IL-4, IL-10, CD39, and CD5-like molecule (CD5L) and play immunomodulatory roles.

The pathogenicity of Th17 cells is closely related to autoimmune diseases, and this pathogenicity depends on IL-6, IL-1β, and IL-23.^101^ Single-cell RNA-sequencing showed that pathogenic Th17 cells showed increased proinflammatory genes such as *IL17a*, *IL17f*, and *IL23r*, and decreased immunomodulatory genes such as *IL4*, *IL10*, and *Cd5l*.^101–103^ Th0 cells did not express IL-1R or IL-23R. During Th17 differentiation, RORγt can promote the expression of IL-1R and IL-23R.^104,105^ The IL-6-STAT3 axis can also inhibit the expression of IL-1R and the IL-23R negative regulator Forkhead box O1.^106^ Subsequently, IL-1β and IL-23 stimulate Th17 cells to become pathogenic. IL-1β regulates the expression of RORγt^107^; IL-23 can activate JunB and inhibit SOCS, subsequently activating STAT3 expression,^108^ and it can also inhibit B lymphocyte maturation protein 1 (Blimp-1) to inhibit IL-10.^109^ In addition, IL-1β and IL-23 can promote the expression of the transcription factor Bhlhe40 through the IL-1β-Bhlhe40 axis and the IL-23-Satb1-Bhlhe40 axis, inducing the expression of granulocyte-macrophage colony-stimulating factor (GM-CSF).^110,111^ GM-CSF is one of the key factors in Th17 pathogenicity. GM-CSF acted on APCs and promoted the secretion of IL-6 and IL-23, which promoted the positive feedback of Th17 differentiation^101^ (Figure 2).

Th17 cells are closely related to AIH. Several studies have shown that RORγt, IL-17A, IL-6, IL-22, and IL-23 levels are significantly increased in PBMCs of AIH patients.^32–36^ Wu et al^37^ found that IL-17A expression was significantly elevated in both AIH patients and Con A-induced AIH mice and that T-cell-immunoglobulin and mucin domain 3 (Tim-3) may inhibit Th17 cell-related expression and AIH through the p38-MKP-1 pathway. T-cell differentiation requires energy and metabolites, in which glutamine metabolism plays an important role.^112^ Yu et al^113^ found that targeted suppression of glutamine metabolism could reduce Th1 and Th17 differentiation and inhibit Con A-induced AIH. Zhao et al^38^ found an increased frequency of CCR4^+^CCR6^+^ Th17 in PBMCs of AIH patients. Th17 cells were significantly infiltrated in the portal tracts and lobular areas, and the levels of Th17-related transcription factors and cytokines were significantly increased in the liver.^38^ Cell experiments confirmed that IL-17 could promote the expression of IL-6 in human hepatoma HepG2 cells through the MAPK pathway and that positive feedback promoted the differentiation of Th17 cells.^38^ CCN1 is an extracellular matrix-associated protein.^114^ IL-17 can promote the expression of CCN1, and CCN1 can promote the production of IL-6 and induce Th17 differentiation.^115^ Jiang et al^116^ found that CCN1 expression was increased in the liver of AIH patients and that CCN1 could increase IL-6 expression through the α_6_β_1_-PI3K-Akt-NF-κB axis. CD39 is an enzyme that catalyzes the hydrolysis of extracellular ATP/ADP to produce AMP, which is then converted to immunosuppressive adenosine through CD73.^117^ CD39^+^ Th17 cells can produce adenosine to exert immunosuppressive effects and reduce its pathogenicity. Studies have shown that CD39^+^ Th17 cells, immunosuppressive adenosine, and A2A adenosine receptor expression are decreased in adolescents with autoimmune liver diseases such as AIH.^95^ CD39 promotion can be modulated by aryl hydrocarbon receptor (AhR) signaling. AhR interacts with its ligand AhR nuclear transporter to promote downstream gene expression, and HIF-1α can bind to AhR nuclear transporter to inhibit AHR signaling.^118^ Studies have shown that the expression of HIF-1α is increased in Th17 cells in AIH, which inhibits AhR signaling and CD39 mRNA expression, and HIF-1α silencing can partially restore CD39 levels.^119^

### Tregs and AIH

Treg cells are important immunosuppressive cells, which are divided into tTregs and peripheral-induced Treg cells according to their origin.^120^ tTregs mature after positive and negative selection in the thymus and can be enriched by TCRs that recognize self-antigens; peripheral-induced Treg cells can be differentiated from Th0 cells on exposure to IL-2 and TGF-β.^121^ Treg cells have been identified as CD4^+^CD25^+^Foxp3^+^ T cells, and Foxp3 is a key transcription factor.^122^ Foxp3 is involved in the expression of several key genes, such as *IL2Rα* and *cytotoxic T-lymphocyte-associated antigen-4* (*CTLA-4*), and interacts with other factors, such as GATA3 and runt-related transcription factor 1, to maintain the characteristics of Tregs.^123–125^ Ectopic expression of Foxp3 can enable CD4^+^CD25^−^ conventional T cells to acquire immunosuppressive function.^126^ However, Foxp3 is expressed transiently in Th0 cells following TCR stimulation, but it is undeniable that not all Th0 cells undergo Treg differentiation and acquire immunosuppressive function.^127^ Studies have shown that the differentiation and function of Treg cells are dependent on Foxp3 expression and Treg-specific hypomethylation, which are 2 independent processes.^128,129^ In addition, various posttranslational modifications of proteins can modulate Foxp3 transcriptional activity, such as phosphorylation, ubiquitination, and acetylation.^130,131^

Treg cells exert immunosuppressive effects mainly by secreting inhibitory cytokines and expressing inhibitory cell-surface molecules and competitive inhibitory cytokines.^132^ The inhibitory cytokines secreted by Tregs are mainly TGF-β and IL-10. TGF-β plays an important role in the maintenance of Treg cells. As mentioned, TGF-β participates in Treg differentiation. Tregs produce TGF-β, which can upregulate Foxp3 expression through the TGF-β-Smad pathway and establish positive feedback to support Treg differentiation^133^; IL-10 inhibits various cytokines, such as IL-2, IFN-γ, and GM-CSF, to inhibit Th1 and Th17 differentiation.^134,135^ Treg cells highly express the inhibitory receptor CTLA-4, which plays an important role in immunosuppression. CTLA-4 acts on its ligands CD80/CD86 expressed by APCs, inhibiting the costimulatory interaction between CD80/CD86 and CD28 and thereby preventing the activation of T cells.^136^ Tregs can also mediate the apoptosis of Tim-3^+^ T cells through Galectin-9 binding to Tim-3.^137^ IL-2 is an important cytokine involved in the proliferation and differentiation of effector T cells (Teffs). Treg cells express CD25 (IL-2Rα), which can competitively consume IL-2 and exert immunosuppressive effects.^138^ In addition, low-dose IL-2 preferentially activates Treg differentiation due to the high IL-2 affinity of CD25^+^ Treg cells, and Treg cell-based IL-2 therapy may play a role in the treatment of autoimmune diseases^139^ (Figure 2).

Current studies have shown that the frequency and function of Treg cells change significantly in AIH. These changes are conflicting in different studies, but it is plausible that altered Tregs are not sufficient to control inflammation in AIH.^10,140^ Adoptive Treg transfer and increased Treg/Teff ratio attenuate hepatic hepatitis in different AIH models.^33,39–41^ The percentage of Treg cells in PBMCs of ANA/SMA^+^ or LKM1^+^ AIH patients was significantly lower than that of normal controls, and even lower at diagnosis than in remission stage, but the inhibitory function to IFN-γ was maintained.^42^ In addition, the suppressive effect of Treg cells on CD8^+^ T cells was significantly weakened, and CD8^+^ T cells were hyper-responsiveness at diagnosis. Tregs inhibited the proliferation of CD8^+^ T cells and induced the production of IL-4 by CD8^+^ T cells at remission.^43^ Another study reported that in addition to a reduction in numbers, Treg cells from AIH patients had a diminished ability to produce TGF-β.^44^ In AIH-SLE/MTCD, the number and function of Foxp3^+^ Treg were abnormal, and CD4^+^CD25^−^IFN-γ^+^ and CD4^+^CD25^−^IL-17^+^ T cells were significantly increased.^28^ CD127 is usually presented on activated Teffs, while Tregs are usually CD127^low/−^.^10^ Peiseler et al^45^ reported that compared with active AIH patients, the frequency of CD4^+^CD25^high^CD127^low^ Tregs decreased in PBMCs of AIH patients in remission. Longhi and colleagues found a decrease in CD127^−^ Tregs and an increase in CD127^+^ Tregs in AIH. CD127^−^ Tregs showed a suppressive effect on Teffs, whereas CD127^+^ Tregs showed increased TNF-α over IL-10 production and increased TLR4 expression.^141^ The expression of CD39 in Treg cells is decreased in AIH, and silencing estrogen receptor-α or AhR repressor can promote AhR signaling and upregulate immunosuppressive adenosine.^119^ In addition, the levels of Tim-3 in Treg cells Galectin-9 and CD4^+^ Teff cells were significantly decreased in AIH.^142^ A lower Treg frequency and Treg/Teff ratio may be associated with a higher recurrence rate of AIH. As a clinical standard treatment for AIH, corticosteroids and azathioprine have significant inhibitory effects on Tregs.^45,143^ Taubert et al^143^ found a reduction in the frequency of portal vein Tregs in AIH patients undergoing treatment. In addition, patients with biochemical remission have a higher Treg/Teff ratio than those who do not achieve remission. Liberal et al^144^ found reduced responsiveness of Treg cells to IL-2 and reduced IL-10 production in AIH-1. Diestelhorst et al^145^ showed that AIH patients who did not achieve biochemical remission with corticosteroid therapy had associations with IL-2 deficiency and impaired Tregs. Based on the high affinity of Tregs for IL-2, Buitrago-Molina and colleagues treated EAH mice with complexed IL-2/anti-IL-2. After treatment, the frequency of Tregs and the ratio of Tregs/CD4^+^ T cells in the PBMCs, spleen, and liver were significantly increased, and the ALT level was significantly decreased.^146^ In addition, IL-2 therapy significantly improved the suppressive effect of corticosteroids on Tregs. The study by Lim and colleagues demonstrated the therapeutic potential of low-dose IL-2 in AIH. low-dose IL-2 treatment increased the frequency of Tregs and the sensitivity of Tregs to IL-2 in patients with refractory AIH, and there was no significant change in other immune cells.^147^ Overall, restoring Treg frequency and function will be a feasible measure for the treatment of AIH.

### Th9 cells and AIH

IL-9 was initially considered to be a cytokine produced by Th2 cells, but a unique CD4^+^ T-cell subset producing IL-9 was subsequently found and identified as Th9 cells.^148^ Th9 differentiation requires balanced stimulation by TGF-β and IL-4, and PU.1 and interferon regulatory factor 4 are key transcription factors in Th9 differentiation.^149^ Studies have shown that both PU.1 and interferon regulatory factor 4 can directly act on the *IL9* promoter to promote IL-9 transcription.^150,151^ Balanced costimulation by IL-4 and TGF-β is crucial in Th9 differentiation.^152,153^ As previously mentioned, IL-4 and TGF-β are key cytokines required for Th2 and Treg cell differentiation, respectively. TGF-β can activate PU.1 and induce Th9 differentiation^154^; TGF-β can also activate Smad2 and Smad3 through the TGF-β-Smad axis and then interact with interferon regulatory factor 4 to activate the promoter of *IL9*.^155,156^ However, TGF-β activates Foxp3, an inhibitor of Th9 differentiation, causing Th0 cells to differentiate into Tregs.^157^ IL-4 activates STAT6, leading to the production of IL-9 in Th2 and Th9 cells.^158^ STAT6 can also inhibit Foxp3 and relieve the negative effect of TGF-β on Th9 differentiation.^159^ The IL-2-STAT5 axis is also involved in Th9 differentiation, and the Th9 promoter has STAT5-binding sites.^160,161^

**FIGURE 3:**
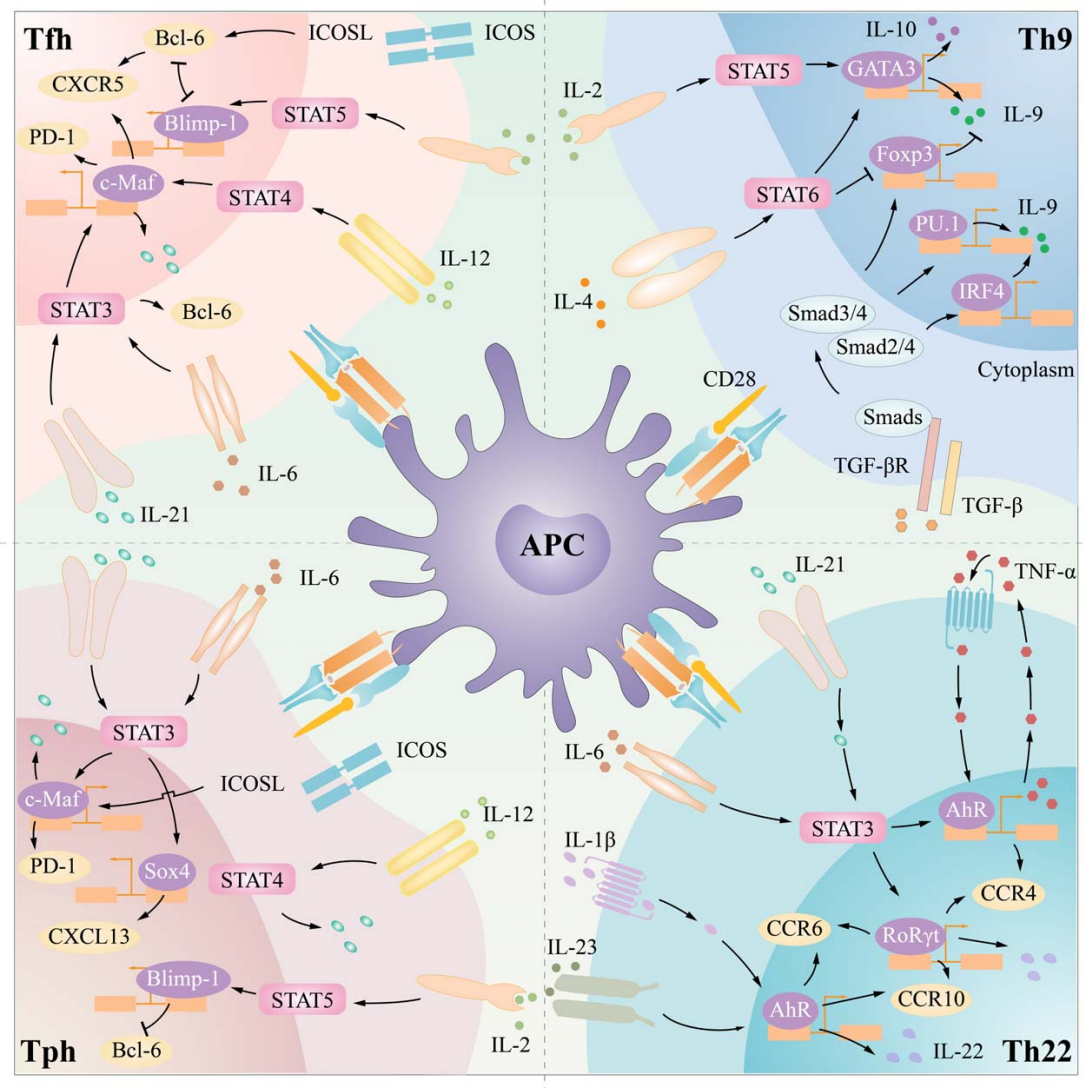
Processes of Th9, Th22, Tfh, Tph differentiation. pMHC-TCR and CD28-CD80/CD86 costimulation and various cytokines induced different differentiation of Th0. Balanced stimulation with IL-4 and TGF-β induced PU.1 and IRF4 expression and Th9 differentiation. IL-21, IL-6, IL-1β, IL-23, and TNF-α stimulated AhR expression and Th22 differentiation. IL-12, IL-21, IL-6, and ICOS promote c-Maf and Bcl-6 expression and PD-1^+^CXCR5^+^ Tfh differentiation. IL-21, IL-6, IL-12, and ICOS promote c-Maf, Blimp-1 and Sox4 expression, and Tph differentiation. Abbreviations: AhR, aryl hydrocarbon receptor; APC, antigen-presenting cell; Bcl-6, B-cell lymphoma 6; Blimp-1, B lymphocyte maturation protein 1; c-Maf, cellular musculoaponeurotic fibrosarcoma; CXCL13, CXC chemokine ligand 13; CXCR5, CXC chemokine receptor 5; Foxp3, forkhead box P3; GATA3, gata-binding protein-3; ICOS, inducible costimulator; IRF4, interferon regulatory factor 4; JAK, Janus kinase; MHC, major histocompatibility complex; RORγT, retinoid-related orphan receptor-gamma t; Sox4, SRY-related high mobility group box 4; STAT, signal transducer and activator of transcription; Tfh, T follicular helper; Tph, T peripheral helper.

IL-9 and IL-10 are the main effector molecules of Th9 cells. IL-10 is a potent anti-inflammatory factor. IL-9 binds to its receptor IL-9R to exert its effects.^162^ IL-9R is composed of an α chain and γ chain, and the α chain specifically binds to IL-9, while the γ chain is a common chain that is also present in IL-2, IL-4, IL-7, IL-15, and IL-21 receptors.^46^ After IL-9 binds to IL-9R, JAK is activated, which subsequently leads to the activation of downstream STAT1/3/5.^163,164^ IL-9 has pleiotropic effects on different immune cells, such as mast cells, T cells, and B cells, and is involved in various immune and autoimmune diseases^165^ (Figure 3).

At present, there is still a lack of research on Th9 cells in AIH. However, several studies have reported that Th9 cells and IL-9 play pathogenic roles in multiple sclerosis, SLE, experimental autoimmune encephalomyelitis, and other autoimmune diseases.^166–168^ Qin et al[169] explored the level of Th9 cells in liver fibrosis caused by HBV or chronic hepatitis B. In this study, the levels of Th9 cells and IL-9 were significantly increased in both patients and mice with liver fibrosis. Moreover, after IL-9 was inhibited, liver fibrosis and the levels of proinflammatory Th1 and Th17 cells were decreased in mice.^169^ This study may reveal a partial role for Th9 cells in AIH, as liver fibrosis and cirrhosis are also later features of AIH.^2^ However, more relevant studies are still needed to confirm the specific role of Th9 cells in AIH. As mentioned, the anti-inflammatory cytokine IL-10 is also produced by Th9 cells.

### Th22 cells and AIH

Th22 cells were identified in 2009 as a CD4^+^ T-cell subset characterized by IL-22 secretion.^170^ Th22 cells also secrete IL-26, TNF-α, and IL-13 but do not express IFN-γ, IL-4, or IL-17.^171^ In addition, Th22 cells express the chemokine receptors CCR4, CCR6, and CCR10.^172^ Th22 differentiation requires the involvement of multiple cytokines and transcription factors. The cytokines involved in inducing Th22 differentiation mainly include IL-6, TNF-α, IL-1β, IL-21, and IL-23.^173^ TGF-β is an inhibitor of Th22 differentiation.^173^ AhR is a major transcription factor regulating Th22 differentiation, and T-bet and RORγt are also involved in regulating Th22 differentiation.^174,175^

Th22 cells exert their effects by secreting IL-22. IL-22 receptor is composed of IL-22R1 and IL-10R2.^47^ IL-22R1 has a high affinity for IL-22 and is not expressed in lymphoid tissues, so IL-22 can only indirectly regulate immune cells through signaling.^48,49^ IL-22 binds to IL-22R and activates JAK and tyrosine kinase to subsequently exert its effects through JAK-STAT, extracellular signal-regulated kinase 1/2, and other signaling pathways.^50,173,176^ Th22 cells play both anti-inflammatory and proinflammatory roles in tumors, cardiovascular diseases, and immune diseases^171^ (Figure 3).

Both IL-10R2 and IL-22R1 are expressed in hepatocytes, and IL-22 binds to its receptor to induce the activation of JAK-STAT, MAPK, p38, and other pathways.^49,177,178^ The results of current studies on Th22 cells and IL-22 in AIH are contradictory. Behfarjam et al^179^ described elevated AHR mRNA levels in the serum of AIH patients. Liang et al^33^ found that the serum IL-22 level was significantly increased in activated AIH patients and positively correlated with ALT and aspartate aminotransferase levels in patients. Experimental AIH mice showed an increased hepatic Th22 cell frequency, increased serum IL-22 levels, and sustained replication of AhR.^33^ The levels of Th22 cells and IL-22 were found to decrease after the use of immunosuppressive drugs.^33^ However, Zenewicz et al^180^ found that IL-22 reduced the destruction of hepatocytes by the immune response during acute liver inflammation and that IL-22 deficiency sensitized hepatocytes in mice to liver inflammation. Pan et al^181^ found that hepatocyte IL-22 overexpression could treat Con A-induced liver injury by activating STAT3 and promoting the expression of the antiapoptotic proteins Bcl-2 and Mcl-1. A single-cell RNA-sequencing analysis revealed that IL-22 expression was elevated in Con A-induced AIH and was further increased after treatment with the AhR agonist 2,3,7,8-tetrachlorodibenzo-p-dioxin.^182^ However, ALT levels decreased after 2,3,7,8-tetrachlorodibenzo-p-dioxin treatment. Elevated AhR expression also led to decreases in the Th17/Treg ratio and the activation of CD4^+^ T cells, CD8^+^ T cells, B cells, and NK cells.^182^ Th22 cells may be a potential therapeutic target in AIH, but more studies are needed to clarify the exact mechanism by which Th22 cells are involved in the pathogenesis and progression of AIH.

### Tfh cells and AIH

In addition to secreting proinflammatory/anti-inflammatory factors, CD4^+^ T cells also support B-cell proliferation and differentiation, promoting humoral immunity.^183^ Tfh cells are a subset of CD4^+^ T cells present in the lymph nodes and spleen that participate in the germinal center (GC) formation, antibody class switching, and B-cell differentiation and maturation.^184^ Tfh cells characteristically express CXC chemokine receptor 5 (CXCR5), inducible costimulator (ICOS), and B-cell lymphoma 6 (Bcl-6) and highly express programmed cell death protein 1 (PD-1).^185^ Bcl-6, IL-6, and IL-21 are essential factors in Tfh differentiation.^186–188^

Tfh cells are regulated by different factors during differentiation, migration, and interaction with B cells. Tfh differentiation depends on IL-21, IL-6, and CD28-CD80/CD86 costimulation.^189,190^ IL-21 is the main cytokine produced by Tfh cells and is also an important factor regulating Tfh differentiation.^191^ IL-21 can induce the differentiation of Tfh cells and can directly regulate the proliferation, maturation, and differentiation of B cells by binding to IL-21R in B cells.^190^ Both IL-21 and IL-6 can activate cellular musculoaponeurotic fibrosarcoma (c-Maf) through the JAK-STAT3 pathway, and c-Maf can upregulate IL-21 transcription.^189,192,193^ c-Maf is also involved in CXCR5 expression.^194^ Tfh differentiation was shown to be significantly impaired in mice deficient in IL-21 or IL-6.^195,196^ In addition, IL-12 and IL-27 are positive regulators of Tfh differentiation, while IL-2 and IL-7 are negative regulators of Tfh differentiation.^197–201^ CD28 costimulation is necessary for Tfh differentiation, and CD28^−/−^ mice were found to have a Tfh differentiation deficiency and impaired GC development.^202,203^ CTLA-4 inhibits CD28 stimulation by binding to CD80/CD86, thus inhibiting Tfh differentiation.^204,205^ Bcl-6 is an essential transcription factor regulating Tfh differentiation.^188^ At present, the pathway by which Bcl-6 promotes Tfh differentiation is still unclear, but studies have shown that ectopic expression of Bcl-6 in CD4^+^ T cells could lead to increased expression of CXCR5, PD-1, and ICOS^188,194^; in addition, the absence of Bcl-6 inhibited Tfh differentiation.^206^ Bcl-6 is highly expressed in Tfh cells, while Blimp-1 is highly expressed in CD4^+^ Teffs.^207^ Bcl-6 and Blimp-1 are antagonistic to each other. Bcl-6 inhibits Blimp-1 and transcription factors of Th1, Th2, and Th17 cells (T-bet, GATA3, and RORγt) to maintain Tfh differentiation^208,209^; Blimp-1 downregulates Bcl-6 and PD-1, inhibiting Tfh differentiation.^209,210^ ICOS is induced by CD28-CD80/CD86 costimulation and plays an important role in Tfh differentiation by binding to its ligand ICOSL.^211^ Studies have shown that ICOS-induced PI3K plays an important role in Tfh differentiation.^212,213^ ICOS can enhance the expression of Bcl-6, c-Maf, IL-21, CXCR5, and CD40L and inhibit Blimp-1 and CCR7.^214^ Activin A can also activate CXCR5 and inhibit CCR7 to support Tfh differentiation.^215^

The localization of Tfh cells to B-cell follicles requires high expression of CXCR5 and ICOS and low expression of CCR7.^51,52,212^ CXCR5-expressing cells can be attracted by the ligand CXCL13 expressed in B-cell follicles, mediating the migration of T cells into the interior of B-cell follicles and T–B-cell interactions.^52,53^ CCR7 encourages T cells to stay in the T-cell region of GCs.^51^ ICOS mediates Tfh cell migration by binding with ICOSL on follicular bystander B cells.^212^

Signaling lymphocyte activation molecule (SLAM)/SLAM-associated protein (SAP) and ICOS/ICOSL signaling play an important role in T–B-cell interactions. SLAM receptors colocalize with TCRs in activated T cells, and on activation, a tyrosine residue in the cytoplasmic SLAM tail is phosphorylated and bound to SAP, subsequently activating SAP and triggering complex signal transduction.^54,55^ SAP deficiency can lead to impaired GCs and inhibition of B-cell proliferation.^56^ SLAM/SAP signaling is also involved in the regulation of ICOS. ICOS/ICOSL signaling is important in maintaining Tfh polarization.^216^ Downregulation of CXCR5 and upregulation of CCR7 induced by ICOS inhibition could result in the reversal of Tfh polarization and exit from B-cell follicles.^214^ Due to the importance of humoral immunity in adaptive immunity, there is no doubt that Tfh cells play an important role in immune function and autoimmune diseases (Figure 3).

Tfh cells and IL-21 are closely related to AIH. Ma et al^217^ found that hypergammaglobulinemia in Chinese AIH patients was accompanied by a marked increase in the PD-1^+^ICOS^+^IL-21^+^ Tfh cell population in PBMCs and abnormal activation of B cells. Tfh cells and IL-21 have been positively correlated with serum effector B cells, IgM, and IgG in AIH patients. Abe and colleagues highlighted the role of IL-21 in AIH. An increase in the IL-21 expression was observed in patients with different stages of AIH, regardless of whether the patient was in remission or experiencing severe AIH, and was positively correlated with the severity of AIH.^218^ IL-21 is also positively correlated with the CCR6-CCL20 axis and CXCR3-CXCL9 axis and participates in the migration of immune cells expressing CXCR3 or CCR6.^218^ Aoki et al^219^ generated a lethal AIH model by removing the thymus of PD-1-deficient neonatal mice. In this model, ICOS^+^IL-21^+^ Tfh differentiation was significant in the GCs in the spleen, and inhibition of ICOS or IL-21 reduced Tfh differentiation. In addition, the authors identified CCR6^+^ Tfh cells and revealed the importance of the CCR6-CCL20 axis in the migration of CCR6^+^ Tfh cells to the liver.^219^ Kimura et al^220,221^ found that the level of CCR7^−^ Tfh cells was elevated in PBMCs of AIH patients with low levels of IgG, which may be helpful for early diagnosis of AIH. Ma et al^222^ found that Lactobacillus enhanced the therapeutic effect of prednisone in both AIH patients and experimental AIH mice. Prednisone + Lactobacillus could significantly improve the levels of IL-21 and Tfh cells in PBMCs of AIH patients and reduce the expression of IL-21, Bcl-6, and CXCR5 mRNA in the liver in mice.^222^ In summary, Tfh cells may be an indicator to predict the progression and treatment of AIH, but more studies are necessary to analyze Tfh cells and the functions of Tfh cell subsets (Tfh1, Tfh2, and Tfh17) in the liver of AIH patients.

### Tph cells and AIH

In addition to Tfh cells in secondary lymphoid tissues, other CD4^+^ T cells in peripheral tissues contribute to B-cell activation and antibody production; these cells are known as Tph cells.^223^ Tph cells were originally identified in rheumatoid arthritis and shown to express the Tfh hallmarks IL-21, ICOS, c-Maf, and high levels of PD-1.^224^ However, unlike Tfh cells, Tph cells express low levels of Bcl-6 and do not express CXCR5, which makes Tph cells a specific CD4^+^ T-cell subset.^224^

Tph cells express low levels of Bcl-6 and high levels of Blimp-1.^225^ Studies have shown that Bcl-6-deficient and CXCR5-deficient mice can still produce specific antibodies.^226,227^ The independence of Tph cells from Bcl-6 highlights the importance of Bcl-6 in Tfh differentiation. High levels of Bcl-6 promote CXCR5 expression and Tfh differentiation, as well as Tfh cell migration to B-cell follicles and GC formation.^185,228^ Tph cells with low levels of Bcl-6 cannot enter B-cell follicles, and they express chemokine receptors such as CCR2, CCR5, and CCR9, which promote Tph cell migration into different peripheral tissues.^229,230^ SRY-related high mobility group box 4 is another transcription factor critical for Tph differentiation, which is stimulated by STAT3 and promotes CXCL13 production.^57,231^ CXCL13 recruit CXCR5^+^ B cells to induce plasmacytoid differentiation in situ^232^ (Figure 3).

Tfh and Tph cells can both regulate B-cell proliferation and differentiation through c-Maf and IL-21. c-Maf can promote the expression of IL-21, and IL-21 can directly regulate the proliferation and differentiation of B cells by binding to IL-21R in B cells.^190,233,234^ Tfh and Tph cells can both regulate B-cell proliferation and differentiation through c-Maf and IL-21. c-Maf can promote the expression of IL-21, and IL-21 can directly regulate the proliferation and differentiation of B cells by binding to IL-21R in B cells.^190,233,234^ Tph cells can promote B-cell differentiation and antibody production in rheumatoid arthritis and SLE, but few studies have been conducted in AIH.^235,236^ Renand et al^237^ found that PD-1^+^CXCR5^−^CD4^+^ T cells were enriched in PBMCs of patients with active AIH or remission and that activation of humoral immunity and antibody production in AIH mainly depended on CD45RA^−^CD27^+^PD-1^+^CXCR5^−^CD4^+^ T cells but not on PD-1^+^CXCR5^+^CD4^+^ T cells. The factors regulating the differentiation relationship between Tph and Tfh cells are not fully understood, and further studies are needed to identify common and specific targets and pathways.

## CONCLUSION

**FIGURE 4:**
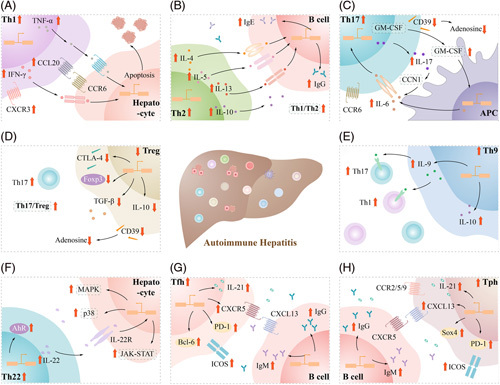
Roles of different CD4^+^ T cells in AIH. (A) Th1 produces IFN-γ and TNF-α, promoting the homing of Th1 to the liver and the apoptosis of hepatocytes. (B) Th2 produces IL-4, IL-5, and IL-13 that promote B-cell differentiation and induce antibody class switching to IgG1 and IgE. The Th1/Th2 ratio is imbalanced in AIH. (C) Th17 cells produce proinflammatory cytokines such as IL-17 and GM-CSF, which increase the positive feedback expression of IL-6 and Th17. CCR6 promotes Th17 migration to the liver. (D) The decrease of Foxp3^+^ Treg in AIH leads to the decrease of TGF-B, IL-10, and CTLA-4 and the imbalance of Th17/Treg ratio. (E) Th9 cells produce IL-9, which acts on IL-9R, activates JAK-STAT1/3/5, and promotes the proinflammatory effect of Th1, Th17, and other immune cells. (F) Th22 produces IL-22, acts on IL-22R in hepatocytes, and exerts effects through JAK-STAT, MAPK, p38, and other pathways. (G, H) IL-21^+^ICOS^+^CXCR5^+^ Tfh and IL-21^+^ICOS^+^CXC13^+^ Tph cells recruit CXCL13^+^ B and CXCR5^+^ B cells, respectively, and promote antibody class switching to IgG and IgM. Abbreviations: Bcl-6, B-cell lymphoma 6; CCL20, chemokine (C-C motif) ligand 20; CCR6, C-C chemokine receptor 6; CTLA-4, cytotoxic T-lymphocyte-associated antigen-4; CXCL13, CXC chemokine ligand 13; CXCR5, CXC chemokine receptor 5; PD-1, programmed cell death protein 1; Sox4, SRY-related high mobility group box 4.

CD4^+^ T cells are unique and important components of adaptive immunity, and different CD4^+^ T-cell subsets play unique roles. In this manuscript, we describe the characteristics of the CD4^+^ Th1, Th2, Th17, Th22, Th9, Treg, Tfh, and Tph T-cell subsets and changes in the frequency and function of CD4^+^ T-cell subsets in AIH (Figure 4). Autoantigens mediate abnormal changes in the frequency and function of these CD4^+^ T-cell subsets in the liver immune microenvironment. CD4^+^ T-cell subsets promote liver autoimmune inflammation and regulate the progression of AIH through cytokine secretion and other mechanisms. Notably, current studies showed some contradictions, such as changes in the frequency and function of Th2, Treg, and Th22 cells in AIH. The factors leading to these contradictions may include genetic factors, epigenetic factors, and environmental factors, leading to different changes in different AIH populations. However, these contradictions seem to be acceptable. No matter how Th2 and Treg change, the overall Th1/Th2 and Teff/Treg ratio are imbalanced, reflecting the imbalance of cellular immunity/humoral immunity and immune promotion/suppression. Th22 and Th9-related studies are still less and their special roles need to be further explored in the pathogenesis of AIH.

As first-line long-term immunosuppressive therapy for AIH has serious side effects, safer targeted therapies are urgently needed. Based on the critical role of CD4^+^ T-cell subsets in autoimmunity, targeting CD4^+^ T-cell subsets seems to be a potential therapeutic approach for AIH. However, due to the effectiveness of clinical first-line treatment, the complexity of autoimmune targets, the side effects of related biological agents and other factors, current clinical progress of targeting CD4^+^ T-cell subsets in the treatment of AIH is still poor. Current studies mainly target TNF-α and IL-2.^238^ Studies have shown that infliximab, an anti-TNF-α monoclonal antibody, can achieve laboratory remission in refractory AIH patients, but some patients develop severe infectious complications.^239^ In addition, ~1 in 120 AIH patients treated with infliximab experienced hepatotoxicity. Therefore, further clinical studies are necessary to evaluate the efficacy and safety of anti-TNF-α in the treatment of AIH.^240^ Encouragingly, low-dose recombinant IL-2 was shown to improve Treg cells and clinical and biochemical parameters with minimal side effects in 2 AIH patients.^241^ This makes it possible to target Tregs, while more studies addressing how to increase the specificity and persistence of IL-2-stimulated Tregs are needed.

It should be pointed out that this manuscript is limited to reviewing the changes of CD4^+^ T-cell subsets related transcription factors, cytokines, chemokines, related ligands, and signaling pathways in AIH, but does not explore their specific mechanisms in AIH. In addition, because the acquisition of intrahepatic CD4^+^ T cells in AIH patients requires an invasive liver biopsy, there are few studies on the function of intrahepatic CD4^+^ T cells in AIH patients. In addition to CD4^+^ T cells, other immune cells, such as CD8^+^ T cells, B cells, macrophages, and NK cells, also play an important role in the regulation of the AIH liver immune microenvironment. These interactions constitute a complex immune regulatory network, and more in-depth studies are needed to identify these interactions and potential cross-talk mechanisms and reveal the complex immune dysregulation in AIH.

In conclusion, the abnormal frequency and function of CD4^+^ T-cell subsets play key roles in AIH. The differentiation and function of CD4^+^ T cells are affected by genetic factors, genetic epigenetic factors, and environmental factors. Current studies revealed abnormal changes in several CD4^+^ T-cell subsets in AIH, while the specific mechanisms of several CD4^+^ T-cell subsets in AIH are still unclear. CD4^+^ T cells and other immune cells constitute a complex AIH immune regulatory network. Based on the in-depth understanding of the frequency, function, migration, antigen specificity, and plasticity of CD4^+^ T-cell subsets in AIH, further studies are needed to explore the interactions and cross-talk mechanisms between CD4^+^ T-cell subsets and other immune cells. These studies will help determine the complex mechanisms and key targets of CD4^+^ T-cell subsets involved in the progression of AIH, providing theoretical guidance in identifying new biomarkers of AIH and discovering more effective and safer therapeutic targets to replace the current long-term immunosuppressive therapy, which is known to have serious side effects.
